# Underestimated Subsequent Sensorineural Hearing Loss after Septicemia

**DOI:** 10.3390/medicina59111897

**Published:** 2023-10-26

**Authors:** Chun-Gu Cheng, Yu-Hsuan Chen, Yin-Han Chang, Hui-Chen Lin, Pi-Wei Chin, Yen-Yue Lin, Ming-Chi Yung, Chun-An Cheng

**Affiliations:** 1 Department of Emergency, Taoyuan Armed Forces General Hospital, Taoyuan 32549, Taiwan; doc50015@yahoo.com.tw (C.-G.C.);; 2 Department of Emergency Medicine, Tri-Service General Hospital, National Defense Medical Center, Taipei 11490, Taiwan; 3 Division of Chest Medicine, Department of Internal Medicine, Cheng Hsin General Hospital, Taipei 11220, Taiwan; anemia0829@gmail.com; 4 Department of Psychology, National Taiwan University, Taipei 10621, Taiwan; 5 School of Nursing, College of Nursing, Taipei Medical University, Taipei 11031, Taiwan; cecilia@tmu.edu.tw; 6 Department of Nursing, Ministry of Health and Welfare, Hua-Lien Hospital, Hualien 97061, Taiwan; 7 Department of Cardiovascular Surgery, Taiwan Adventist Hospital, Taipei 10540, Taiwan; 8 Department of Neurology, Tri-Service General Hospital, National Defense Medical Center, Taipei 11490, Taiwan

**Keywords:** septicemia, hearing loss, apoptosis, complication

## Abstract

*Background and Objectives*: Hearing loss after septicemia has been found in mice; the long-term risk increased 50-fold in young adults in a previous study. Hearing loss after septicemia has not received much attention. The aim of this study was to assess the relationship between septicemia and subsequent hearing loss. *Materials and Methods*: Inpatient data were obtained from the Taiwan Insurance Database. We defined patients with sensorineural hearing loss and excluded patients under 18 years of age. Patients without hearing loss were selected as controls at a frequency of 1:5. The date of admission was defined as the date of diagnosis. Comorbidities in the 3 years preceding the date of diagnosis were retrieved retrospectively. Associations with hearing loss were established by multiple logistic regression and forward stepwise selection. *Results*: The odds ratio (OR) for the association between sepsis and hearing loss was 3.052 (95% CI: 1.583–5.884). Autoimmune disease (OR: 5.828 (95% CI: 1.906–17.816)), brain injury (OR: 2.264 (95% CI: 1.212–4.229)) and ischemic stroke (OR: 1.47 (95% CI: 1.087–1.988)) were associated with hearing loss. *Conclusions*: Our study shows that hearing loss occurred after septicemia. Apoptosis caused by sepsis and ischemia can lead to hair cell damage, leading to hearing loss. Clinicians should be aware of possible subsequent complications of septicemia and provide appropriate treatment and prevention strategies for complications.

## 1. Introduction

Septicemia survivors can experience varying degrees of disability and problems that can change their lives. The modern definition of sepsis is an abnormal host response to infection, followed by life-threatening organ dysfunction. Septicemia is a serious infectious condition that can cause cardiac events, neurologic events and mortality [1,2,3,4]. Septicemia has a high incidence of critical care hospitalization [5]. Sepsis has a complex pathophysiology involving multiple immune and non-immune mediators. It is now believed that immune system hyperactivation and cascades of inflammation are often accompanied by immunosuppression during the initial stages of sepsis. Neutrophils are crucial in the pathophysiology of severe sepsis. Recent studies have shown a clear link between the neutrophil death process and the emergence of organ dysfunction in sepsis. During sepsis, spontaneous apoptosis of neutrophils is inhibited, and neutrophils may undergo other types of cell death [4]. Approximately 14.1% of people aged 20 to 69 in the United States have hearing loss [6], which is caused by multiple processes and causes complications, leading to social withdrawal and reduced quality of life. A previous study found that septicemia was linked to hearing loss in a mouse model of sepsis [7]. Subsequent hearing loss after septicemia deserves study.

Causes of hearing loss include aging, genetic effects, injury, ototoxic drugs, infection, cardiovascular and neurologic diseases, immune diseases and loud voices. In animal studies, inflammation induces apoptosis of cells in the organ of Corti and the supporting cells of the lateral wall, especially changes in type I fiber cells and inner hair cells of the spiral ligament, and stimulates glutamate overload in inner hair cells [7,8]. The labyrinthine artery branches from the anterior inferior cerebral artery, supports the inner ear and is sensitive to ischemic stimuli.

Limited hair cell recovery after injury in mammals results in persistent hearing impairment [9]. Clinicians must understand the underlying factors and develop optimal prevention strategies to reduce hearing loss. A previous study investigated hearing loss after septicemia for a long-term period [10]. We used the Taiwan National Health Insurance Research Database (NHIRD) to check the related factors of hearing loss for a 1-year period. We used multivariate logistic regression to reveal the association between septicemia and subsequent hearing loss.

The aim of our study was to assess whether sepsis is associated with subsequent hearing loss in Taiwan. We also examined other risk factors for subsequent hearing loss. Early detection and timely treatment of complications are important to reduce hearing deficits.

## 2. Materials and Methods

Taiwan has had National Health Insurance since 1995, a government-backed health care system that covers approximately 99% of Taiwan’s 23 million citizens. All healthcare providers must type computer data to pay for insurance. The Taiwan NHIRD contains all outpatient and inpatient medical insurance payment records. There are up to 5 International Classification of Disease, Ninth Revision, Clinical Modification (ICD-9-CM) inpatient diagnosis and procedure codes and 3 outpatient ICD-9-CM diagnosis and procedure codes. The data contain each patient’s age, gender, comorbid conditions, hearing loss codes and times of admission and discharge.

**Figure 1:**
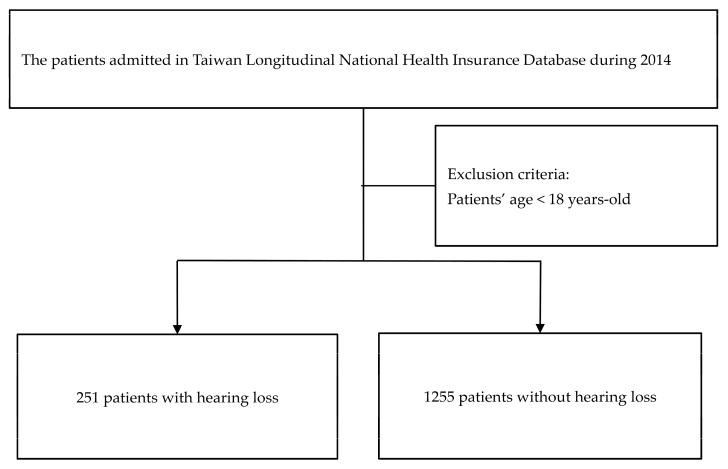
Flow chart of this study.

The dataset used in this study comes from the Taiwan Longitudinal Health Insurance Database (LHIRD), which randomly selects patients from the population. We identified patients with hearing loss diagnosis codes collected between 1 January 2014 and 31 December 2014, and we excluded patients under the age of 18 years and those who were deaf before sepsis admission. The time of admission for hearing loss was defined as the date of diagnosis in the study group. Five controls were frequency-matched to each case according to age and gender. This does not distinguish between risk factors and the order of hearing loss that occurs during hospitalization. Our aim was to identify risk factors affecting subsequent hearing loss; thus, comorbidities were retrieved before the diagnosis date of hearing loss. The flow chart is shown in Figure 1. This study was approved by TSGHIRB-B-105-11.

We used ICD-9-CM codes to find hearing loss codes and diagnoses of comorbidities in the NHIRD. We defined transient ischemic deafness as 388.02 and sensorineural hearing loss as 389.1X in the inpatient dataset. Hearing loss comorbid disorders mapped by ICD-9-CM codes included septicemia (038, 003.1, 036.1), hypertension (401–405), diabetes mellitus (250), chronic kidney disease (stage 2–4, codes 581, 582, 583, 585, 588; end-stage renal disease, codes 585.6, 586, V42.0), hyperlipidemia (272), autoimmune disorders (710), anemia (280, 285), hematological disorders including coagulopathies and platelet disorders (286–287), coronary artery disease (410–414), ischemic stroke (433–437), meningitis (320–322, 013.0, 036.0, 053.0, 047), head injuries (800, 803–805, 850–854) and encephalopathy (293, 348.3, 780.01, 780.09).

Continuous variables are shown as the mean ± standard deviation and were evaluated using Student’s *t* test, while categorical variables are shown as percentages using the chi-square (X^2^) test. Multivariate logistic regression with forward stepwise selection was used to assess correlates of hearing loss and ischemic stroke. The statistical significance was set as *p* < 0.05. The statistical analysis was performed with SPSS version 21 software.

## 3. Results

**Figure 2:**
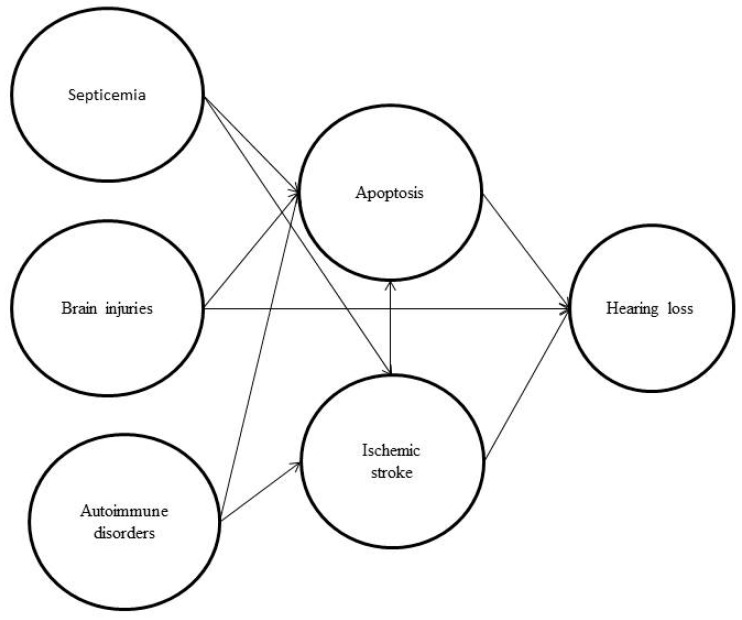
The potential pathway of hearing loss after sepsis showing how septicemia, brain injuries, autoimmune disease and ischemia will induce apoptosis, modified from [7,11,12,13].

We retrospectively surveyed 251 hearing-impaired patients as the study group and 1255 non-hearing-impaired patients as the control group. Table 1 shows the demographic variables for the hearing loss and control groups. There were more instances of septicemia, hypertension, brain injuries, coronary artery disease, ischemic stroke, meningitis and hematological disorders (platelet and coagulopathy disorders) in the hearing loss group (Table 1). Figure 2 shows the potential pathway of hearing loss after sepsis.

Our study found an association between sepsis and hearing loss with an odds ratio (OR) of 3.052 (95% confidence interval (CI): 1.583–5.884). Other factors included autoimmune disorders (OR: 5.828 (95% CI: 1.906–17.816)), brain injuries (OR: 2.264 (95% CI: 1.212–4.229)) and ischemic stroke (OR 1.47 (95% CI: 1.087–1.988)) (Table 2).

We also assessed ischemic stroke-related factors and revealed risk factors including septicemia (OR: 3.566 (95% CI: 1.227–10.36)), hyperlipidemia (2.437 (95% CI: 1.399–4.247)), hypertension (6.686 (95% CI: 4.649–9.616)), coronary artery disease (6.21 (95% CI: 4.34–8.884)) and hematological disorders (3.363 (95% CI: 2.159–5.24)) (Table 3).

## 4. Discussion

The finding of our study was that sepsis is significantly associated with subsequent sensorineural hearing loss. It has been associated with hearing loss from pre-existing sepsis, ischemic stroke, autoimmune disease and brain injury. When a patient has a severe infection, healthcare providers need to pay more attention to comorbidities such as cerebrovascular attack, autoimmune disease and traumatic brain injury to prevent hearing loss.

A previous study in Thailand showed a relative risk of 1.8 (95% CI: 1.0–3.2) for factors associated with hearing loss in infants, including sepsis, low birth weight, APGAR (appearance, pulse, grimace, activity, respiration) score < 6 in 5 min, craniofacial anomalies and ototoxic exposure [14]. Sepsis has been found to induce apoptosis in multiple organs, leading to multiple organ failure [4,15]. Hearing loss after sepsis is caused by immune-induced apoptosis or microthrombosis with destruction. Glutamine stimulation in the radial dendrites induces apoptosis in Deiters cells, Claudius cells and Hensen’s cells and induces vacuolization of inner ear cells and immunohistochemical effects through a cecal ligation puncture in a mouse study [7].

Common causes of hearing loss include noise, ototoxic drugs, sepsis, electrolyte abnormalities, dehydration and malnutrition [16]. Sepsis is caused by an infection anywhere in the body. Bacteria or viruses with toxins can spread to other parts of the body through the blood. This inflammation can lead to blood clots and affect the oxygen supply to organs, leading to failure. Unstable blood pressure in patients with sepsis can cause insufficient blood flow, which in turn affects hearing. Sensorineural hearing loss is associated with infectious and metabolic diseases, with changes in pure tone audiometry of more than 30 dB. Common mechanisms associated with hearing loss in the hospital setting include microemboli, perfusion abnormalities, hypercoagulability and ototoxic drugs. The use of endotracheal, nasotracheal and positive pressure face or nasal masks may impair the function of the Eustachian tube, leading to ascending bacterial colonization, changes in middle ear pressure, middle ear obstruction, and fluid accumulation, leading to conductive hearing loss. Toxins can reduce extreme blood flow, which can lead to organ or tissue damage [17].

Sepsis with encephalopathy may lead to microcirculation insufficiency, which reduces inner ear perfusion and leads to radial dendritic edema [18]. Fibroblast formation, immune dysregulation and hypercoagulation lead to microvascular thrombosis in patients with sepsis [19]. Sepsis with hemodynamic instability and impaired cerebrovascular autoregulation can induce hypohemodynamic stroke.

Autoimmune diseases are pathological conditions caused by the immune system misrecognizing self-antigens, resulting in damage and destruction of host tissue. These diseases affect different organs and systems, including the blood, joints, skin and muscles. In autoimmune diseases, innate immune cells fail to function properly: they are either abnormally activated or physically disabled. As an important innate immune cell, neutrophils are affected by the microenvironment of different autoimmune diseases due to their short lifespan and diverse death modes. They are associated with the neutrophil death pathway; delayed or abnormal apoptosis may contribute to the development of autoimmune disease [11]. Autoimmune diseases promote an inflammatory response to nerve demyelination and hypoperfusion. Immune complex deposition in the auditory artery leads to the release of oxidative molecules from hair cells and spiral ganglion damage leading to blood flow with hypoxia [20]. Autoimmune disorders, such as systemic lupus erythematosus (SLE), sicca syndrome and psoriatic arthritis, can predispose patients to or be associated with autoimmune inner ear disease. Anticardiolipin antibodies play a pathogenic role in hearing loss in SLE. Cardiolipin and M3 muscarinic receptor antibodies were found in patients with sicca syndrome. B2-glycoprotein antibodies associated with thromboembolic events and minor vasculitis of the inner ear arterial microvessels may lead to hearing loss [21,22]. One-third of cases of autoimmune inner ear disease are also associated with systemic autoimmune disorders. Middle-aged females have the highest prevalence. Antibodies against the antigens of the inner ear cause antibody-mediated cytotoxic or complex-mediated immune injury, which is the pathogenesis of hearing loss in animal models. Previous studies have shown that the surrounding tissue of the endolymphatic sac contains components for the immunological reaction. It produces an autoimmune reaction in sensitive cells in the inner ear, which can circulate into the cochlea. The damage includes lesions of the organ of Corti, retrograde neural degeneration to the spiral ganglion, endolymphatic hydrops, stria vascularis dystrophy, neofibroosteogenesis in the basal turn of the cochlea, and fibrosis of the endolymphatic sac and lymphocytes in the labyrinthine membrane compartment [21]. A previous study found that stroke patients receiving steroids and predisposed to vasculitis had a risk of sudden sensorineural hearing loss with an HR of 5.14 [23]. Our study found similar results, with subsequent hearing loss being significantly associated with autoimmune disorders.

Head trauma and brain injury are often associated with underlying damage to the inner ear as well as blunt or penetrating head trauma and hemorrhagic lesions of the auditory cortex [16]. Traumatic brain injury (TBI) occurs when an external physical force strikes the head with sufficient force to cause damage to the brain and can have long-term consequences, including vision difficulties, cognitive deficits, headache, sleep disturbances and posttraumatic seizures. Disruption of normal brain function leads to a cascade of effects including molecular and anatomical changes, persistent neuronal hyperexcitability, neuroinflammation and neuronal loss. Destructive processes occurring at the cellular and molecular levels lead to inflammation, oxidative stress, calcium dysregulation and apoptosis. Vascular injury, ischemia and loss of blood–brain barrier integrity lead to the destruction of brain tissue. Excitotoxicity increases intracellular calcium influx, and the generation of free radicals leads to the opening of mitochondrial permeability transition pores, resulting in the release of cytochrome c into the cytoplasm to induce apoptosis [12]. Hearing impairment in brain-injured patients results from direct nerve damage or diffuse axonal damage. Conductive hearing loss involves damage to the ossicles caused by trauma. During the acute phase of brain injury, an anti-inflammatory response protects against brain damage, followed by an inflammatory response during the chronic phase, leading to cell death and delayed demyelinating changes [24]. The risk of long-term hearing loss was associated with brain injuries with a hazard ratio of 2.125 [25]; our study showed a similar finding.

Previous studies have found that multiple risk factors, including hypertension, diabetes, dietary risk scores, current smoking status, alcohol consumption, psychosocial stress, depression and cardiac distress, contribute to stroke in multiple countries [26]. Apoptosis is the most common form of programmed cell death in multicellular organisms and can be triggered by intrinsic or extrinsic pathways. The activation of immune cells during inflammation caused by cerebral ischemia may release a variety of factors that trigger neuronal cell death through the extrinsic apoptotic pathway, including pro-inflammatory cytokines [13]. The initial morphological changes of apoptosis include cell shrinkage and cytoplasmic condensation, followed by the disruption of the nuclear envelope and the formation of apoptotic bodies, all of which occur in the absence of any inflammatory response. These features of apoptosis have also been observed in postischemic stroke neurons. Ischemic stroke is caused by insufficient circulation to the inner ear due to atherosclerosis and hemodynamic hypodynamics. A previous study found that circulatory disease plays an important role in hearing loss. The labyrinthine artery, which is a branch of the anterior inferior cerebellar artery, can lead to hearing loss associated with cerebellar infarction. Because the cochlea is supplied by the labyrinthine artery, it is highly sensitive to poor perfusion. Loss of the spiral ganglion and flattening of the organ of Corti were found after ischemia using guinea pigs. They were associated with high-frequency auditory changes, and the duration of ischemia leads to hair cell damage with more vulnerability in the outer hair cells [27]. A previous rat study demonstrated cellular damage with apoptosis on the cuticular plate of outer hair cells in a carotid ischemia model [28]. Previous studies have shown that stroke patients are at risk for sudden hearing loss with an adjusted hazard ratio of 1.71 for the long term and 5.65 within one year of follow-up [22]. However, our study found that the risk of ischemic stroke was relatively low. The underlying reason was that patients with ischemic stroke may have cognitive impairment but no hearing complaints. Moreover, in previous studies, short- and long-term ischemic stroke were associated with sepsis [1,2,3]. Thus, we investigated factors similar to those in the previous study. Patients with sudden sensorineural hearing loss were more likely than controls to have vertebrobasilar insufficiency (OR, 1.76; 95% CI, 1.02–3.04) in a previous study [29]. However, our subgroup analysis of vertebrobasilar artery syndrome (ICD-9-CM of 435.0, 435.1, 435.3) showed that subsequent hearing loss was not significant. The underlying cause is that patients had brainstem infarctions, and the conditions tended to be too severe to perform hearing function surveys to develop the diagnosis.

Men with strenuous work and noise exposure have a higher risk of brain damage and hearing loss from pneumococcal meningitis [30]. Men (61%) subsequently developed hearing loss in a previous study group. Adult hearing loss is common and strongly associated with age (60–69 years: OR, 39.5; 95% CI, 10.5–149.4); however, male sex (OR, 1.8; 95% CI, 1.1–3.0) was also an important risk factor [6]. For men, diabetes, hypertension, smoking and ≥2 major CVD risk factors were associated with hearing loss. For women, diabetes, smoking and ≥2 major CVD risk factors were significant risk factors [31]. Children and elderly patients are vulnerable to injury from hearing loss caused by pneumococcal meningitis. This affects elderly adults after age 55 with an age-related effect [32]. Exposure to loud noise can tear the hairs or nerve cells in the cochlea. Aging immune systems are weaker and vulnerable to the lasting effects of sepsis. However, there were higher case–fatality rates in elderly patients with septicemia, ischemic stroke or traumatic brain injury that prevented us from tracing these patients in later stages, which suggests that advanced age was not associated with hearing loss in this study.

Some infectious diseases with high fever, such as meningitis, can damage the cochlea by cochlear ossification and hearing impairment [16]. A previous study found a link between deafness in children and meningitis. Approximately 7% of school-age survivors of bacterial meningitis develop hearing loss [33]. Our univariate analysis found an OR of 2.064 for the association of meningitis with hearing loss. The ability of meningitis to be related to septicemia (crude OR: 11.956 (95% CI: 6.338–22.551), *p* < 0.001) was a potential reason for the loss of importance of meningitis after multivariate adjustment. Some ototoxic drugs, such as aminoglycoside antibiotics, certain chemotherapy drugs and loop diuretics, can damage the inner ear. Meningitis in children was treated with aminoglycosides such as gentamycin, which is ototoxic and causes hearing loss in approximately 2.3% of cases [34]. However, third-generation cephalosporin is used to treat adult meningitis and has fewer toxic effects on the ear. Furthermore, adults with mature immune competence had fewer sequelae in hearing loss in our study.

Hyperlipidemia is associated with ischemic stroke; however, it was not related to adult deafness in our study. The reason may be that the patients with hyperlipidemia were treated appropriately according to the guidelines. The risk of hearing loss in diabetes mellitus in American and Korean women had an OR of 1.9 and 1.4, respectively [35]. But our study did not find risk associated with diabetes mellitus; the potential reason was a higher proportion of hearing loss in males. Loud noise exposure has a risk of hearing loss with an OR of 2.4 in males [6]. The association between iron deficiency anemia and hearing loss had an OR of 2.41 (95% CI: 1.90–3.01) among adults with iron deficiency anemia after adjusting for sex in adults from the US [36]. Lower hemoglobin levels result in an impaired oxygen-carrying capacity. Iron deficiency leads to the degradation of lipid saturases and desaturases, which impair energy production by producing myelin. Reactive thrombocytosis due to iron deficiency anemia increases ischemic risk. However, our study showed that anemia is not associated with subsequent hearing loss. Because the diagnosis of anemia in the NHIRD lacks a degree of anemia influencing hearing function, hemoglobin in hearing loss needs to be surveyed in the future.

Treatment can sometimes help preserve some hearing function. Prophylactic topical anti-inflammatory therapy (such as TNF-alpha neutralizers) should be discussed in systemic inflammation to prevent sensorineural hearing loss [8]. Identifying the source and supplying effective antibiotics can also prevent hearing loss. Dextrans and vasodilator prescriptions can improve circulation to the inner ear. A previous study showed that antiplatelet therapies, beta-blockers and statins can prevent sepsis-induced ischemic stroke [37,38,39]. A vaccine can prevent bacterial meningitis [40]. Steroids are also used to lessen neurological damage and prevent long-term problems such as blindness and hearing loss after bacterial meningitis. Autoimmune-related hearing loss is reversible after immunomodulatory therapy. Plasmapheresis can be managed as an alternative or an adjunct therapy. If treatment fails, an operative cochlear implant can improve hearing, although this alternative is not cheap.

Sensorineural hearing loss is associated with infectious and metabolic diseases and occurs primarily in patients with multisystem failure and septic shock. Unstable blood pressure in patients with sepsis can cause insufficient blood flow, which can affect hearing in intensive care units [16]. According to animal studies, apoptosis caused by sepsis and ischemia can lead to hair cell damage, leading to hearing loss. Air pollution was related to sudden hearing loss related to oxidative stress and immune and infectious diseases in a previous study [41]. The immune and circulatory systems play an important role in subsequent sepsis-induced hearing loss, and a possible pathway is shown in Figure 2. Further research is needed to better understand the potential links between sepsis and hearing loss, and whether screening and treatments for sepsis in adults could be clinically meaningful for patients with hearing loss. Physicians need good treatments for sepsis, autoimmune disease and ischemic stroke, as well as strategies to prevent brain damage from occurring. Preventing hearing loss can enhance communication skills, reduce psychological burden and improve quality of life. Previous studies have focused on single risk factor, and studies about combined factors have not been conducted. This paper provides a preliminary beginning. Prospective studies can address how baseline septicemia and other conditions inducing apoptosis affect long-term auditory function, and the clinical implications will be better understood in the future.

This study has some limitations. First, there was a lack of height, weight and noise data; family history; and severity information from audiometry and otological exams in the payment claim dataset. Patients with severely deteriorated life expectancy, bed-ridden patients or ventilation-dependent patients could not undergo hearing function tests, which may potentially underestimate the number of hearing loss patients. Second, hearing loss in people over the age of 55 is age-related. Our study group was relatively older, with a mean age of 60 years, and 43% of patients were older than 65 years. We need to survey young people to confirm our research. Third, we were unable to examine cerebrovascular status during routine hospitalization. Arterial maps from computed tomography or magnetic resonance imaging are required from registry data to determine blood flow associated with hearing loss. Fourth, the claim data included admission and discharge data, but the time from septicemia to hearing loss in intensive care was not available. A register study is needed for proof.

## 5. Conclusions

This study explored whether sepsis is associated with subsequent hearing loss. Sepsis and ischemia-induced apoptosis are associated with hearing loss resulting from hair cell damage. Healthcare professionals must be aware of factors associated with hearing loss such as sepsis, autoimmune disease, ischemic stroke and brain injury. Hearing function must be considered in patients after hospitalization for sepsis, with early diagnosis and appropriate prevention. A prospective evaluation is needed to confirm our findings.

## Figures and Tables

**Table 1:** Study participant baseline characteristics.

	Hearing Loss (251)	Hearing Loss Free (1255)	*p*
Age	60.17 ± 17.99	59.22 ± 17.39	0.443
Gender (male)	153 (61%)	165 (61%)	1
Septicemia	18 (7.2%)	24 (1.9%)	<0.001 *
Hyperlipidemia	16 (6.4%)	82 (6.5%)	0.926
Diabetes mellitus	52 (20.7%)	227 (18.1%)	0.328
Hypertension	111 (42.2%)	456 (36.3%)	0.019
Autoimmune disease	7 (2.8%)	6 (0.5%)	<0.001 *
Chronic kidney disease			0.706
Stage 2–4	17 (6.8%)	71 (5.7%)	
End-stage renal disease	5 (2%)	20 (1.6%)	
Brain injuries	16 (6.4%)	34 (2.7%)	0.003 *
Coronary artery disease	81 (32.2%)	328 (26.1%)	0.046
Ischemic stroke	89 (35.5%)	310 (24.7%)	<0.001 *
Meningitis	37 (14.7%)	97 (7.7%)	<0.001 *
Anemia	22 (8.8%)	106 (8.4%)	0.869
Hematological disorders	54 (21.5%)	191 (15.2%)	0.014 *

* *p* < 0.05.

**Table 2:** Related factors with their odds ratios for patients with subsequent hearing loss vs. the control patients in Taiwan.

	Univariate Odds Ratio	*p*	Multivariate Odds Ratio	*p*
Age	1.003 (95% CI: 0.995–1.011)	0.432		
Gender (male)	1 (95% CI: 0.757–1.32)	1		
Septicemia	3.962 (95% CI: 2.117–7.417)	<0.001 *	3.052 (95% CI: 1.583–5.884)	0.001 *
Hyperlipidemia	0.974 (95% CI: 0.56–1.694	0.926		
Diabetes mellitus	1.183 (95% CI: 0.844–1.658)	0.328		
Hypertension	1.389 (95% CI: 1.056–1.828)	0.019 *		
Autoimmune disease	5.972 (95% CI: 1.99–17.923)	0.001 *	5.828 (95% CI: 1.906–17.816)	0.002 *
Chronic kidney disease				
Normal	reference			
Stage 2–4	0.787 (95% CI: 0.292–2.118)	0.635		
End-stage renal disease	0.958 (95% CI: 0.314–2.917)	0.939		
Brain injuries	2.445 (95% CI: 1.328–4.502)	0.004 *	2.264 (955 CI: 1.212–4.229)	0.01 *
Coronary artery disease	1.374 (95% CI: 1.005–1.805)	0.047		
Ischemic stroke	1.675 (95% CI: 1.255–2.235)	<0.001 *	1.47 (95% CI: 1.087–1.988)	0.012 *
Meningitis	2.064 (95% CI: 1.376–3.097)	<0.001 *		
Anemia	1.041 (95% CI: 0.644–1.684)	0.869		
Hematological disorders	1.527 (95% CI: 1.089–2.141)	0.014 *		

* *p* < 0.05.

**Table 3:** Ischemic stroke-related factors.

Related Factors	Odds Ratio	*p*
Septicaemia	3.566 (95% CI: 1.227–10.36)	0.019 *
Hyperlipidaemia	2.437 (95% CI: 1.399–4.247	0.002 *
Hypertension	6.686 (95% CI: 4.649–9.616)	<0.001 *
Coronary artery disease	6.21 (95% CI: 4.34–8.884)	<0.001 *
Hematological disorders	3.363 (95% CI: 2.159–5.24)	<0.001 *

* *p* < 0.05.

## Data Availability

The datasets used in the current study are available from the corresponding author upon reasonable request.
